# Review of Novel and Emerging Proximal Soil Moisture Sensors for Use in Agriculture

**DOI:** 10.3390/s20236934

**Published:** 2020-12-04

**Authors:** Marcus Hardie

**Affiliations:** Tasmanian Institute of Agriculture, University of Tasmania, Hobart, TAS 7000, Australia; marcus.hardie@utas.edu.au

**Keywords:** matric potential, capacitance, soil moisture probes, dielectric constant, soil humidity, soil water FDR, TDR

## Abstract

The measurement of soil moisture in agriculture is currently dominated by a small number of sensors, the use of which is greatly limited by their small sampling volume, high cost, need for close soil–sensor contact, and poor performance in saline, vertic and stony soils. This review was undertaken to explore the plethora of novel and emerging soil moisture sensors, and evaluate their potential use in agriculture. The review found that improvements to existing techniques over the last two decades are limited, and largely restricted to frequency domain reflectometry approaches. However, a broad range of new, novel and emerging means of measuring soil moisture were identified including, actively heated fiber optics (AHFO), high capacity tensiometers, paired acoustic / radio / seismic transceiver approaches, microwave-based approaches, radio frequency identification (RFID), hydrogels and seismoelectric approaches. Excitement over this range of potential new technologies is however tempered by the observation that most of these technologies are at early stages of development, and that few of these techniques have been adequately evaluated in situ agricultural soils.

## 1. Introduction

Knowledge of soil moisture is important for supporting agricultural production, catchment hydrology, flood forecasting, landslide prediction and other ecosystem services [1,2,3]. Globally, agriculture is the largest water user accounting for approximately 70% of total water consumption [4]. Global demand for diminishing water resources has triggered renewed interest in the development of proximal soil moisture sensors for improved management of irrigation and soil moisture in agriculture. Proximal soil sensors are defined as being in contact with, or within proximity to the soil (<2 m). Proximal sensors are usually classified as being (i) *in-situ*, stationary or point scale, including both invasive or buried sensors, or (ii) noninvasive sensors which may operate on or near the ground surface including being attached to a vehicle to generate ‘maps’ of soil moisture variability [3,5]. Use of non-proximal platforms such as drones, aircraft and satellites are not in the scope of this review, see [3,6,7,8].

An extensive range of proximal soil moisture sensors have been commercialized for use in agriculture. Yet despite the efforts of government agencies, and private consultants, remarkedly few farmers use sensors for monitoring soil moisture or scheduling irrigation. Literature on the adoption of soil moisture sensors is scarce, studies report adoption rates of around 8–13% in Australia, [9], 18% in Washington State, USA, [10] and 3–4% in Southern Alberta, Canada [11]. Poor adoption seems to be related to a combination of operational and soil constraints rather than issues with sensor accuracy. Operational constraints include cost, data volume and interpretation, poor soil–sensor contact, small measurement volume, lack of portability and installation hassle, creation of in-field navigation hazards, and risk of damage to infield electronic components by machinery, stock and pests [3,12,13,14]. Soil constraints include potential errors or sensor failure in saline, vertic, ferric, organic or stony soils and in some cases, the need for soil specific calibration to achieve desired accuracy.

Soil moisture monitoring in agriculture is currently dominated by a small number of ‘trusted’ technologies namely, frequency domain reflectometry (FDR) or capacitance, gypsum block sensors, time domain reflectometry (TDR), and in some industries the neutron moisture meter (NMM) and amplitude domain reflectometry (ADR), reviewed in [3,12,15,16]. Examination of the soil moisture sensor market and ‘Agri-Tech’ boom reveals that most of the supposedly ‘new’ soil moisture sensors that have become commercially available in the last 5–10 years are based on pre-existing dielectric techniques (mostly FDR). Techniques which have been genuinely improved in recent years include multi-depth down hole TDR, low cost TDR sensors, and pseudo TDR approaches (ADR etc.). Very few genuinely new methods for measuring soil moisture have been commercialized or adopted for use in agriculture in the last 2 decades. Poor adoption of emerging techniques appears to result in part poor understanding by technologists of the opportunity that some of the emerging sensor technology poses for overcoming current constraints to the use of existing soil moisture sensors. Equally poor adoption results from the limited knowledge and understanding of these emerging techniques. Accordingly, this review was conducted to,

(i) explore the sensor and engineering literature to identify promising new opportunities for the development of soil moisture sensors for use in agriculture,
(ii) identify opportunities to overcome soil and operational constraints to the use of existing soil sensors, through development of new sensing technologies, and
(iii) seek opportunities to bridge the gap between technologists and the soil community who share a common desire for the development of new soil moisture sensing technology.

## 2. Advances in In Situ Invasive Matric Potential Sensors

Matric potential sensors and tensiometers measure the soil matric potential or the amount of suction required to remove water from the soil rather than soil moisture content. As such matric potential sensors are considered a better measure of plant moisture stress than soil moisture content [17]. Matric potential is measured by either tensiometers, gypsum blocks or granular matrix (i.e., watermark) sensors. However, use of tensiometers is greatly restricted by water cavitation around 80–100 kPa, their small sensed area, need for hydraulic connectivity between the sensor and the soil, and difficulties rewetting following cavitation. The Sciroot sensor has sought to overcome soil–sensor connectivity issues by replacing the porous tip with a flexible 100 cm long geotextile wick which is buried within the crop root zone. The ability of the Sciroot sensor to maintain hydraulic connectivity in different soils has not been reported. Limitations with the Sciroot sensor include the limited operating range (0 to −50 kPa), the need for considerable soil disturbance for installation, and difficulty rewetting the geotextile without excavation following cavitation [18].

The use of matric potential sensors including tensiometers, gypsum blocks and granular matrix sensors are limited to non-vertic (non-swelling) soils as these sensors require hydraulic connection between the porous sensor and soil pores. In vertic soils, matric potential sensors often fail as drying causes the soil to break hydraulic connection with the soil. Gypsum block and granular matrix sensors measure the matric potential of the porous material by resistance which is highly sensitive to soil water salinity (i.e., conductivity) including fertilizer application. In order to reduce the error associated with soil salinity and the variable porosity of gypsum block sensors, sensors such as the MPS-2/6 -TEROS 21 (Decagon Devices, Pullman, WA, USA), the EQ3 Equitensiometer (Delta T Devices, Cambridge, UK) and the Tensiomark (ecoTech, BonnGermany) use FDR or impedance to measure the moisture content of a calibrated porous plate for which the water retention characteristic is known [19,20], resulting in greatly improved accuracy, broader operating range (−10 to −50,000 kPa), and lower sensitivity to salinity.

**Figure 1:**
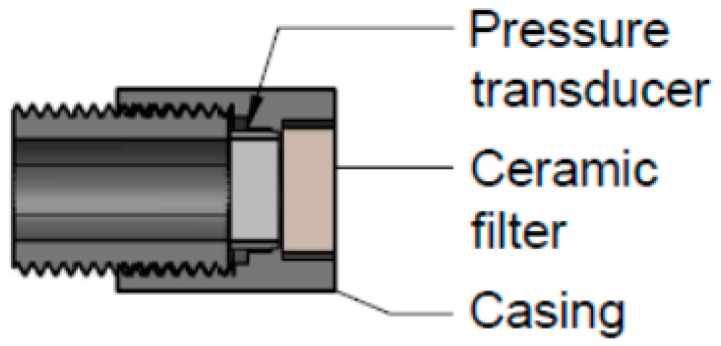
Example of a high capacity tensiometer (HCT) design. Copied with permission from [23].

Prototype high capacity tensiometers (HCTs) operate over the entire plant available water content range (PAWC) from saturation to at least the permanent wilting point (0 to −1500 kPa). The lower operational limit of HCT’s is determined by the air entry value of the filter, the size and smoothness of the measuring chamber, and the aeration and purity of the filling water [21,22]. Ref. [23] tested seven different HCT designs, the best performing design incorporated a small water reservoir of 40 mm^3^ with a flush diaphragm pressure transducer and a kaolin ceramic filter (Figure 1). Their HCT was capable of measuring in excess of −1500 kPa for up to 27 days, and was more accurate and exhibited a faster response than the porous ceramic MPS-2 sensor (Decagon Devices). HCT’s are still at the experimental stage of development and have not been commercialized. Like all tensiometers, their use in agriculture is limited by difficulty purging and re-wetting following cavitation, which in the case of HCTs is especially onerous requiring very high pressure specialized equipment [22,24]. The future use of HCT in agriculture is likely to be limited to research applications unless simple, safe, low cost means of de-airing and preparing the tensiometers can be developed. 

## 3. Advances in In Situ Invasive Soil Moisture Sensors

### 3.1. Dielectric Constant Based Approaches

The majority of commercially available soil moisture sensors or probes (multi-depth sensors) rely on measurement of the soil or electromagnetic relative permittivity [12,25,26]. The dielectric constant (ε*) is a complex phenomenon consisting of real (ε’) and imaginary (ε ”) components [26,27]; as ε* = ε’ – *i* ε”.

The real component ε’ is an indication of the degree of polarization due to water being a polar molecule and thus is directly related to moisture content. The imaginary component ε” accounts for error in the measurement of the dielectric constant associated with the soil, which is related to the loss of energy or dielectric relaxation caused by the attenuation of electromagnetic waves as they pass through the soil [26]. The dielectric constant of water is 80, compared to 1 for air, and around 5 for most soil minerals. The proportion of water to air in a medium greatly affects the real component of the dielectric constant [13,25,28,29], whilst the imaginary component ε” of the constant is influenced by temperature, salinity, clay composition, organic matter and porosity [20,30,31]. Improvements in sensor development has focused on measuring the real part of the permittivity (ε′) using frequencies at or above 100 MHz that minimize, but do not eliminate, the error associated with the contribution of the imaginary component (ε′′) to the dielectric constant [25]. A range of approaches have been developed for measuring soil moisture via the dielectric constant including electrical capacitance or frequency domain reflectometry (FDR), electrical impedance or amplitude domain reflectometry (ADR), time domain transmission (TDT) and time domain reflectometry (TDR) [12,26,32,33,34].

### 3.2. Time Domain Reflectometry (TDR)

TDR sensors are considered a very reliable and accurate method for determining soil moisture. However, their use in agriculture has been limited due to their high cost and need for complex wave form analysis to estimate soil moisture. In recent years, Acclima, Inc. (Meridian, ID, USA) have reduced the size and cost of TDR sensors through the use of cheaper mass-produced electronics including cellular phone components, such that TDR sensors are now competitively priced with high-end FDR sensors. Campbell Scientific (Logan, UT, USA) have also sought to lower the cost of TDR sensors by using transmission line oscillators to create ‘pseudo’ TDR sensors in which the number of reflected voltage pulses are measured rather than conducting complicated waveform analysis of individual reflections [32].

Development of multi depth down-hole TDR probes have been slow to be commercialized. Recently Campbell Scientific Pty Ltd. (Logan, UT, USA) released the SoilVUE10 TDR downhole probe, which measures soil moisture content, permittivity, electrical conductivity (EC) and temperature at nine depths to 1.0 m using a single TDR circuitry unit. Prototype multi-depth TDR sensors have also been developed without great commercial success by [35,36], ESI Environmental Sensors Inc. (Sidney, BC, Canada) and the MP-917 Moisture point TDR system [37,38]. 

### 3.3. Frequency Domain Reflectometry (FDR) and Capacitance

The high cost of TDR sensors has led to the development of alternative lower cost, lower frequency 10–150 MHz, frequency domain reflectometry (FDR) sensors that do not rely on complicated waveform analysis [32]. FDR techniques measure the soil moisture content indirectly by determining the bulk dielectric constant from frequency variations of an electromagnetic pulse propagated into the soil. Due to the lower operating frequencies of FDR, the imaginary component of the dielectric constant can be considerable, such that FDR sensors are more prone to error from soil texture, electrical conductivity, and temperature than TDR sensors [25]. FDR sensors also require careful installation to avoid air gaps between the sensor and the soil [39], and are generally limited to use in non-saline (<1 dS/m) soil [40], and non-vertic soils. In recent years a number of low cost ($5–$50 USD) FDR soil moisture sensors have been developed and commercialized including the DFROBOT SEN0193, Adafruit STEMMA Soil Sensor, Tindie SoilWatch 10, and Vegetronix VH400. For more approaches see [14,40,41,42,43,44,45,46,47,48,49] and others. 

### 3.4. Radio Frequency Identification (RFID)

Ultra-high-frequency radio-frequency identification (UHF RFID) systems operate over a wide range of frequencies from 120 kHz into the microwave bands up to 10 GHz to automatically identify and measure tagged items. RFID provides a very low-cost opportunity for soil moisture monitoring as individual tags (sensors) cost < USD 1 to USD 50; they can be passive (nonpowered) and they can communicate over distances of several meters [50]. Passive RFID tags work by using part of the energy generated by the reader to provide a unique identification, and an analogue voltage output that can be used to power external electronics such as low-power microcontrollers or sensors [51]. 

**Figure 2:**
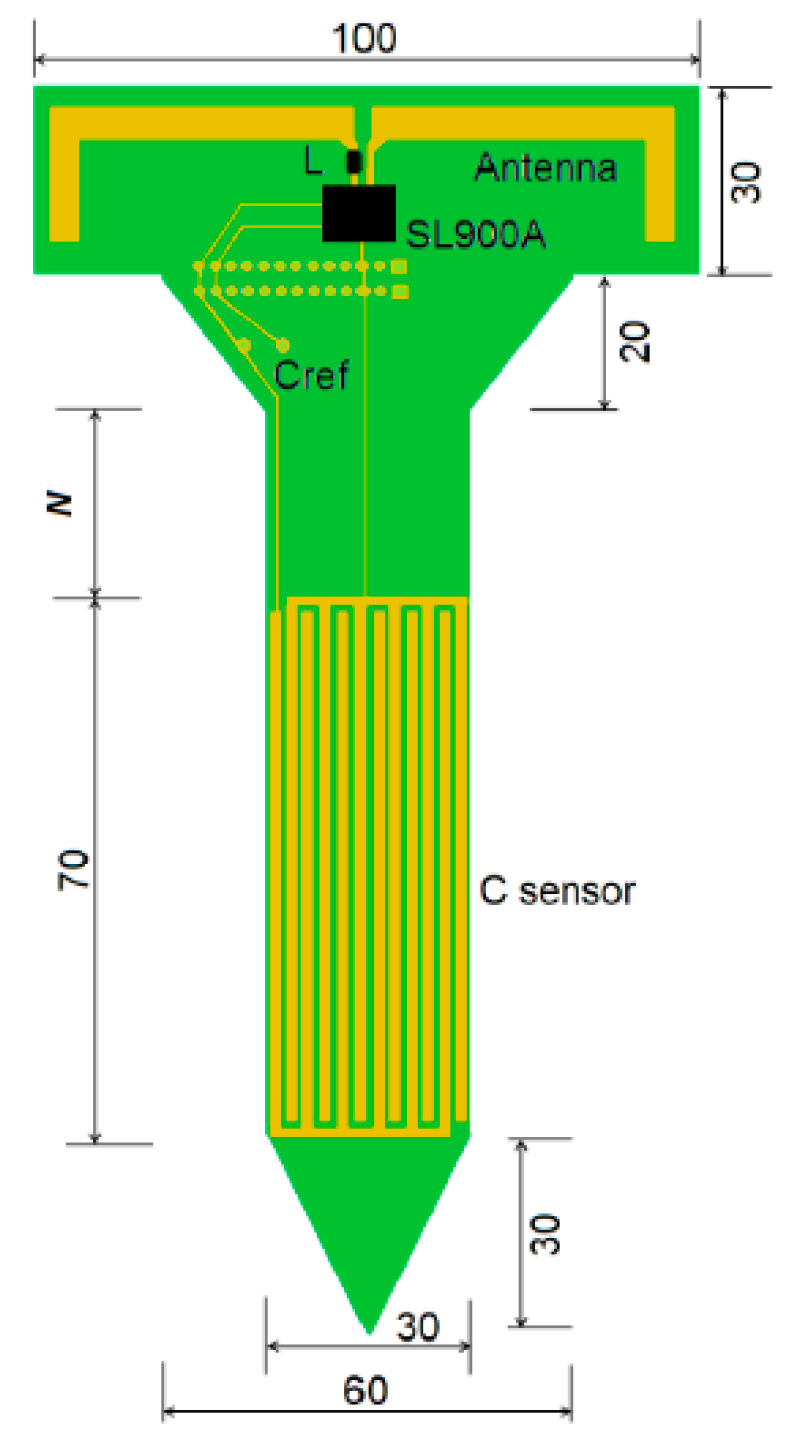
Design of the passive ultra-high-frequency radio-frequency identification (UHF RFID) soil stick sensor (dimensions are in millimeter (mm)). Copied with permission [50].

Over the last 5 years, several novel ways of using RFID tags for measuring soil moisture have been explored. Examples include, [50] who presented two RFID-based soil moisture sensory systems. The first based on a twin tag approach consisted of a reference RFID tag (adhesive labels, FT-G1210) positioned 100 mm above the soil surface and a second tag positioned 12 mm above the soil. Soil moisture content was inferred from the difference in power required to turn on the two tags, which ranged from 7–8 dBm in dry soil, compared to 4–5 dBm in wet soil. Their second approach used a passive UHF chip with a real time clock and an internal temperature sensor, which they reported had a 0.99 correlation with soil moisture (Figure 2). [52] used a SL900A UHF RFID chipset to powerlessly measure soil moisture and temperature using a double-sided interdigital electrode structure to maximize the fringing effect (sensed volume). [51,53] also developed a passive RFID moisture, temperature and relative humidity sensor using a near-field communication (NFC) chip which can be read by an NFC-enabled smart phone. The depth of RFID radio-frequency penetration into soil has not been reported, however use of radiowaves is expected to be greater than that of microwave-based approaches as indicated by radio wave based wireless underground sensor networks (WUSN) which are capable of transmitting several meters through the soil [54,55]. Currently, RFID based approaches offer the only potential for the mass production of very low-cost, passive soil moisture sensors. Use of RFID approaches in agriculture are however limited by the need for a reader to be within <2 m proximity of the RFID tag, and the shallow depth of measurement of RFID approaches. Possible applications of RFID soil moisture sensors may exist in plant nurseries or in immature leafy green vegetable production.

### 3.5. Invasive Open Ended Antenna (Radar) Microwave

Microwaves (300 MHz to 300 GHz) are ideal for remote and proximal sensing of soil moisture, as microwave radiation causes dipole molecules such as water to rotate producing measurable changes in resultant electromagnetic waves [56]. Microwave-based soil sensors have three inherent advantages compared to existing invasive sensors (TDR and FDR), in that they tend to include a greater measurement area (but not necessarily volume), they are not susceptible to error associated with small airgaps between the soil and the sensor [57], and they have multiple modes of deployment including proximal invasive, proximal noninvasive and remote configurations (aircraft and satellite platforms). 

**Figure 3:**
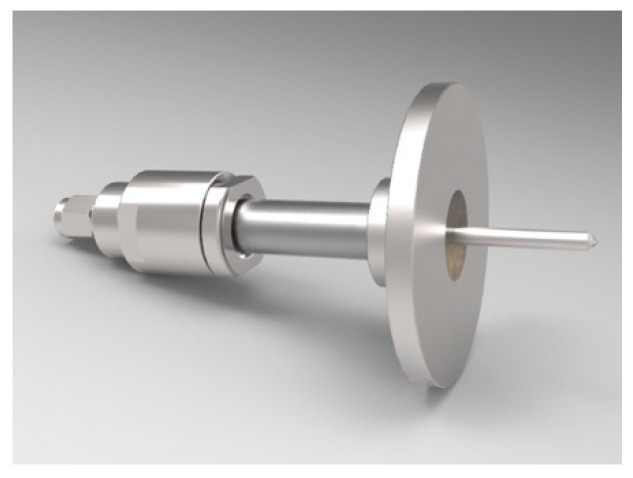
Invasive, open ended microwave antenna. Copied with permission from [39].

Whilst most of the research and development of microwave-based soil moisture sensors is focused on noninvasive mobile approaches (GPR, L-band, etc.), a small number of studies have sought to develop in situ open ended microwave antenna based sensors. Open-ended microwave antenna soil moisture sensors are an emerging area of research in which little information is available about sensor performance, measurement volume, calibration, or the effects of soil properties on error and calibration. Examples include [39] who developed a microwave sensor (Figure 3), which had a correlation between the real permittivity and volumetric soil moisture of 0.99 in three contrasting soil types. [57] also developed an invasive open-ended microwave antenna which consisted of a 16 mm diameter by 170 mm long PVC sealed pipe containing a transmitting (TX) and a receiving (RX) dipole antenna, spaced 50 mm apart. Whist [58] reported that they had developed a compact, low cost and easy to manufacture narrowband open-ended antenna microwave sensor for measuring soil moisture.

### 3.6. In Situ Paired Transceiver Approaches

Experiments using radio, acoustic and seismic wave propagation through soil and rock have shown that wave velocity and signal attenuation are influenced by moisture content [59,60,61,62,63,64,65]. Paired transceiver approaches involve using the velocity or attenuation of either radio, acoustic or seismic waves sent between the paired buried transceivers to infer soil moisture content Paired transceiver approaches present a tantalizing opportunity for the development of soil moisture sensors which have potential to operate over distances of several meters. Furthermore, unlike the NMM and cosmic ray approaches in which results usually consist of information from a number of soil horizons, paired transceiver approaches should in theory be able to operate at discrete depths using the time of flight (ToF) approach which consider the shortest path between transceiver nodes. 

Radiowave approaches have arisen from research on wireless underground sensor networks (WUSN) which seek to wirelessly communicate data between pared buried transceiver nodes via radio frequencies operating between 300 and 950 MHz [54,66,67]. Soil moisture is inferred by measuring either the velocity of wave propagation or time of flight (ToF) through the soil, or by measuring the attenuation of the radio signal between paired transceivers. The ToF approach uses the influence of soil moisture on the dielectric constant of the soil to estimate soil moisture according to;

(1) $$t=\frac{S}{\frac{c}{\sqrt{e_{r}^{\prime }}}}$$

where *t* is the time of flight of the radio frequency signal through the material at distance *S*; *c* is the speed of light in a vacuum, and $e_{r}^{\prime }$ is the real part of the complex relative dielectric constant of the soil. [68] demonstrated they could measure soil moisture over 1–15 m distance using the signal attenuation of radio waves buried at 40 cm depth. Similarly, [69] demonstrated the ability to infer soil moisture by the attenuation of radio waves between transceivers buried at 60 cm depth, located 2–3 m apart.

Acoustic and seismic waves operate in a similar manner to radio wave based WUSN systems in which the propagation of pressure waves through the soil are attenuated by soil properties including soil moisture. Acoustic waves consist of longitudinal waves (P waves) created by pressure oscillations at frequencies between 2000 to 5000 Hz, which travel at the speed of sound 330 m/s in air, 1520 m/s in water, 3000 m/s in clay and 6060 m/s in sand. Seismic approaches differ to radio and acoustic waves in that four different waves are generated by seismic events which provides greater scope for developing relationships between soil moisture and wave characteristics. In addition to P waves, seismic events create secondary shear waves (S waves) which can only travel through solids, and do not travel through liquids or air [70,71,72]. Much of the difficulty with the development of paired transceiver approaches is that wave velocity and signal attenuation are strongly influenced by a range of soil properties other than soil moisture, including density, texture, void ratio or porosity, cementation and electrical conductivity for radio waves, and in the case of S waves effective stress [71,73]. In both seismic and acoustic wave approaches, soil moisture is estimated from the propagation velocity of P waves according to the Brutsaert model which requires knowledge of difficult to measure soil properties including soil porosity, effective pressure and the interstitial parameter Z [62,74,75,76]. Consequently, research effort has been directed to simplification of the Brutsaert model to reduce the unknown parameters, and investigation into the potential to use other waves, specifically the S waves to infer unknown soil parameters or classify soil types for which the unknown parameters are known [59,61,76]. 

Research has demonstrated the feasibility of developing paired transceiver soil moisture sensors. However, considerable uncertainty exists as to which of the three operational wavelengths or approaches (radio, acoustic or seismic) are best suited to soil moisture sensor development or at what scales of operation each of the three wavelengths ideally operate. Equally, the ability of ToF approaches to select for specific soil layers is unproven. Research to date has demonstrated that a range of approaches are able to estimate soil moisture when other soil properties are known. However, development of paired transceiver approaches is limited by the incomplete theoretical understanding of wave propagation in soil, and the extent to which wave velocity and other wave properties are influenced by soil properties other than soil moisture. As such, future research needs to focus on (i) improved understanding of how soil properties influence the velocity and attenuation of different waves within the soil, (ii) use of other waves (non-P waves) or other wave characteristics to derive secondary soil properties and (iii) real world evaluation of approaches in a range of agricultural soils.

### 3.7. Seismoelectric Approaches

Seismoelectric, electrokinetic or electroseismic approaches are an emerging area of geophysics used for noninvasive subsurface exploration for pore fluids such as water, oil and gas. These approaches involve generating a seismic wave which results in the formation of an electromagnetic signal (electrokinetic phenomenon) resulting from pore water flowing from compressed to dilated regions of the soil/rock [77]. Because cations preferentially adhere to capillary walls, the resulting fluid flow separates the cations and anions thus producing an electric dipole causing development of a streaming current and electromagnetic co-seismic field which can be measured at the soil surface using an array of dipole antennas and geophones for measuring the mechanical response [78,79,80,81]. Soil moisture affects both mechanical and electrical properties of soil/rock including seismic velocity, seismic attenuation, electrical conductivity and also the electrokinetic coupling. Consequently, both the coseismic field and interface response properties are influenced by soil water content [82].

Seismoelectric approaches are unique in that they have potential to simultaneously estimate soil porosity, soil moisture content, and hydraulic conductivity over considerable depths [82,83]. The ability of seismoelectric approaches to measure spatio-temporal properties of agricultural soils including soil moisture is largely unknown. Use of seismoelectric techniques in soils is limited to a handful of studies. Ref. [83] demonstrated that the approach could be downscaled to a 25-m-long transect at two sites in the Vosges mountains in which they demonstrated that the electrokinetic coefficient increased with soil moisture content. Whilst [82] reported that the ability of seismoelectric approaches to detect shallow water tables differed with soil type due to the sharpness of the boundary between the partly and fully saturated zones. Given the complexity of the procedure it is unlikely to be able to be routinely used by farmers in the foreseeable future. Future development for near surface applications such as agriculture will require considerable research efforts to better understand seismoelectric processes in shallow variably saturated soils, in addition to deep saturated rocks [77,82,83,84,85,86]. 

### 3.8. Heat Pulse

Heat pulse sensors measure either the thermal conductivity, volumetric heat capacity or the soil thermal diffusivity in response to application of heat, in which moist soils will heat up and dissipate heat slower than dry soils [25,87]. Heat pulse sensors are not affected by salinity or soil temperature [88]. Sensors usually take the form of either a single probe containing both the heating and sensing elements, or dual (multi) probe configurations consisting of a single heater needle surrounded by up to six thermistor needles [3,12,89]. Despite their accuracy, heat pulse sensors have not been widely adopted in agriculture due to their slow response time, high power demand, cost compared to FDR sensors and need for a sophisticated controller to measure heat fluxes [12,28,90].

**Figure 4:**
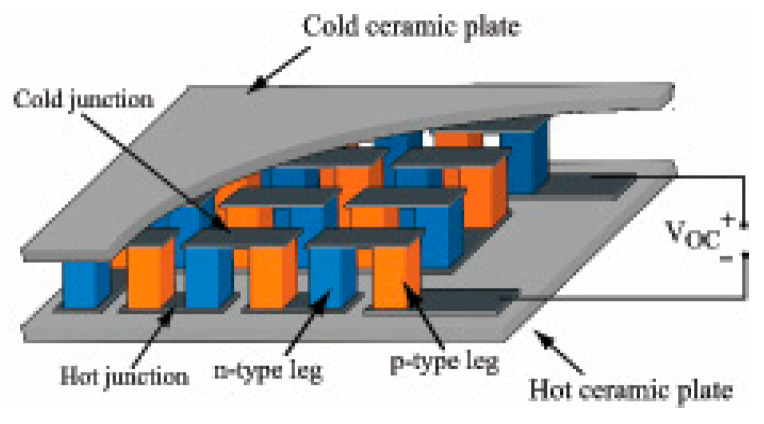
Schematic view of a thermoelectric generator showing the n-type and p-type thermoelectric legs, sandwiched between two ceramic plates. Copied with permission from [89].

Advances in electronics has resulted in the development of a number of low-cost, low-power-use prototype heat pulse sensors [25]. For example, Refs. [91,92] developed a single probe heat pulse sensor based on a single NPN bipolar transistor. They reported that their sensor was 2–6 times more sensitive, and required around one-tenth of the power, compared to traditional thermocouple-based heat pulse sensors. Ref. [89] also developed a low power, highly sensitive single heat pulse matric potential sensor using a nanostructured thermosensitive resistor, powered by an integrated thermoelectric generator (Figure 4). Ref. [93] developed a low-cost, easily manufactured dual probe heat pulse sensor with an on-board microprocessor for controlling and analyzing the heat flux. Their sensor consisted of a 15-resistor-based heater element and a thermistor temperature sensor on an adjacent rod. They reported that their sensor could be manufactured at considerably lower cost, had better precision and required less power than traditional dual heat pulse sensors.

### 3.9. In Situ Fiber Optic Approaches

**Figure 5:**
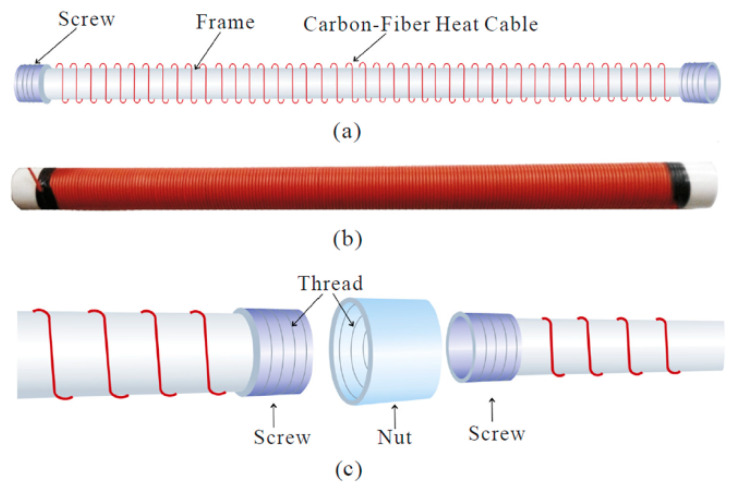
Schematic of a carbon fiber heated down hole soil moisture probe: (**a**) structure, (**b**) the completed probe and (**c**) the screw connection. Copied with permission from [103].

Distributed temperature sensing (DTS) systems measure temperature along a fiber optic cable at the cm scale for distances in the order of kilometers, with a high temporal frequency and great accuracy [94,95,96,97]. Optic fiber-based soil moisture sensors operate by detecting deformation of the optic fiber resulting from either hydration of a hydrophilic (Polyimide) coating on the outside of the optic fiber [98,99], or temperature changes in the soil surrounding the actively heated fiber optic (AHFO) [100,101,102]. The more common AHFO approach involves application of an electrical current through the outer metal sheath of an optic fiber which causes the sheath and surrounding soil to heat up and deform the optic fiber [103,104,105]. Much of the current research on AHFO is focused on use of Fibre Bragg Gratings (FBG) in which variations in the refractive index are inscribed into the core of an optical fiber at prescribed locations to enable precise location and measurement of distortions. AHFO approaches have been used to study dripper wetting patterns in repacked soils [96], along transects at discreate depths [102,106], and as a multidepth soil moisture sensor [104]. Ref. [102] measured changes in soil moisture every 0.25 m along a 147 m long optic fiber cable installed at 5, 10 and 20 cm depths. They reported that the correlation with a commercial soil moisture sensor (Decagon Devices 5TE sensors) ranged from 0.46 to 0.87. Similarly [106] measured changes in temperature every 12 cm along a 300 m fiber optic cable, buried at 0.2 m and 0.4 m depth along a 133 m long transect at a research farm in Spain. Ref. [103] developed a down hole FBG soil moisture sensor with 1.25 cm depth resolution to a depth of 1 m for laboratory studies, and to a depth of 18 m for a field study, by wrapping a carbon-fiber-heated FBG optic fiber around a 5 cm diameter PVC tube (Figure 5). The sensor was installed in an oversized hole then backfilled with coarse sand to prevent damage to the optic fibers. The sensor was able to heat the soil to a distance of up to 7 cm from the probe and achieved a correlation with measured values between 0.93 to 0.94.

The spatially distributed nature of AHFO data appears to be ideally suited to agricultural applications that require knowledge of variations in soil moisture across fields, such as surface or subsurface drip irrigated crops/vines/orchards. In addition, downhole AHFO probes appear to have greater depth resolution and measured soil volume than existing downhole (multidepth) FDR probes. Long term studies of AHFO performance in agricultural systems have not been reported. The use of AHFO approaches in agriculture may however be limited by their susceptibility to damage and deformation of the optic fiber by plant roots, agricultural machinery, and seasonal soil shrink-swelling which may limit the use of AHFO to forms of agriculture with shallow or no tillage, or installation of AHFO beneath the depth of tillage and root growth which isn’t particularly useful for sensing crop moisture stress. Application of AHFO approaches in agriculture are also potentially limited by the need for good sensor–soil contact, need for considerable soil disturbance during installation, slow response time, and complex analysis of large amounts of data [107], and potential need for soil-specific calibrations as indicated by [102,106].

### 3.10. Hydrogels

A small number of studies have sought to use the swelling capacity of hydrogels to measure either soil moisture or matric potential. Hydrogels are highly absorbent hydrophilic polymer chains which can absorb 10–1000 times of their original weight or volume in water over a relatively short period of time [108]. Hydrogel sensors consist of a chemically inert hydrogel polymer, a semiporous membrane/filter/porous plate that prevents migration of the hydrogel into the soil, and a means (mechanical, optical, capacitance) of measuring gel expansion. Hydrogel sensors function in a similar manner to tensiometers in which soil moisture migrates through the semipermeable/porous material causing the hydrogel to expand and contract in equilibrium with the soil matric potential. 

**Figure 6:**
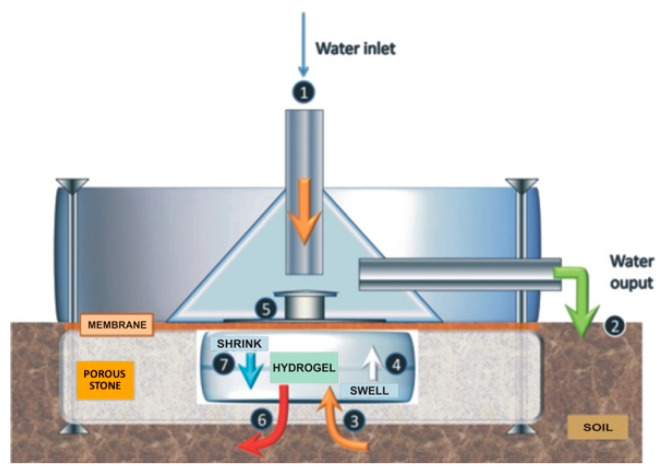
Hydrogel based prototype soil moisture valve (actuator), showing how changes in volume of the hydrogel control the water flow across the system. Copied with permission from [110].

Refs. [109,110] developed a passive (nonpowered) hydrogel-based soil moisture actuator (sensor which can act) for automatically triggering irrigation (Figure 6). Increased soil moisture caused the hygroscopic polyacrylamide hydrogel to swell which pushed on a mechanical rod to cease irrigation. As the soil dried, the hydrogel shrank causing the rod to contract and irrigation to recommence [109]. 

Ref. [110] reported that their cellulose based hydrogel actuator had a response time of 60 min and was independent of soil acidity, and repeatable over a 3-month period. Refs. [111,112] developed a non invasive soil humidity sensor in which moisture content was measured by changes in refractive index of hydrogel coated metal nanostructures as the hydrogel swelled and shrank with soil moisture content. Ref. [112] also reported that on exposure to moisture the resonance position of the spectral peak of their hydrogel sensor shifted from 748 to 720 nm. TOIP Pty. LTD (Moonta, South Australia) have a hydrogel matric potential soil sensor under development which uses capacitance to measure very small increments in gel expansion between a fixed plate and a plate mounted on the hydrogel surface.

Hydrogel-based matric potential sensors represent a new paradigm in soil moisture / matric potential sensors and actuators. The use of hydrogel-based sensors in agriculture is unknown as designs and concepts have only started to be explored in recent years. Potential exist for hydrogel-based sensors to replace water filled tensiometers as they are likely to avoid issues with cavitation and rewetting that limit usability of current tensiometers. However, development of hydrogel-based sensors requires greater understanding of potential environmental influences of temperature, pH and salinity on hydrogel swelling, as well as understanding of potential hysteretic behavior, and hydrogel breakdown over repeated wetting-drying cycles [110].

## 4. Emerging Mobile and Noninvasive Soil Moisture Sensors

Noninvasive soil moisture sensors can be used for both point source and for mobile ‘mapping’ of soil moisture. Mobile, noninvasive approaches have potential to overcome issues with the small measured volume of invasive sensors, by being able to be moved and thus measure larger areas within reasonable timeframes. Non-invasive sensors are also able to operate in stony soils in which installation of invasive sensors is often not possible, and they can operate in vertic soils in which invasive sensors often loose contact with the soil during drying. Difficulties with the use of noninvasive approaches often include issues with limited penetration depth (L band microwave), variable depth of penetration with moisture content (GPR, EMI, Cosmic Ray, L band microwave), difficulty separating response from different soil depths or layers (EMI, Cosmic Ray), the time and hassle involved with conducting surveys (EMI and GPR) and high level of skill required to operate devices and process large volumes of data (GPR).

### 4.1. Cosmic Ray Sensors

Cosmic ray sensors are commercially available, non-invasive (usually) stationary sensors that measure naturally generated neutrons that are produced by cosmic rays passing through the Earth’s atmosphere [113]. They consist of a passive neutron detector placed a few meters above the ground which measures the release (evaporation) of fast neutrons into the air above the soil following neutron collision with hydrogen atoms in the soil [114]. As cosmic ray sensors are noninvasive, they may be suited to stony and vertic soils in which installation of more common types of soil moisture sensors is difficult. Cosmic ray sensors have a very large measurement footprint of around 260–600 m radius [113] which maybe suited to broadscale cropping on uniform soils but are inoperable with the growing trend toward precision agriculture and variable rate irrigation [115,116]. Additional limitations with cosmic ray sensors include their high cost, very large and imprecise measured soil volume, long measurement durations which can be in excess of 4 h, variable depth of measurement which ranges from around 15 cm in wet soils, to approximately 70 cm in dry soils, and difficulty deriving precise calibrations [3,114,117,118]. 

### 4.2. Electromagnetic Induction (EMI)

Electromagnetic induction (EMI) surveys are routinely used in agriculture to map bulk soil variability or changes in soil type, however in recent years they have been increasing used to map variability in soil moisture. EMI surveys are relatively quick to conduct, are non-invasive, have high spatial resolution, and require less specialized skill and knowledge to process and interpret data than other geophysical approaches such as GPR [119,120,121,122,123]. Unlike dielectric or microwave-based based approaches (TDR, FDR, GPR etc.) EMI sensors are not directly sensitive to water content or hydrogen ion content. They respond to the quantity of ions (salt content) in the soil solution, in which increased soil moisture content increases the abundance and mobility of ions and thus increases the apparent electrical conductivity (ECa) [119,124]. 

Much of the recent research effort to improve EMI-based mapping or measurement of soil properties including soil moisture content have focused on the use of (i) multicoil and multifrequency devices [125,126,127], (ii) calibration algorithms [128,129,130], or (iii) inversion approaches for 2D or quasi 3D projection of ECa [131,132,133]. ECa is a bulk response to the proximal environment, incorporating both inherent soil properties (soil minerology and texture), and variable properties (temperature, moisture content, salinity, and soil density) [120,124,134,135]. Consequently, the relationship between ECa and soil moisture content is complex, often co-related with other soil properties, and spatially and temporally unstable [119,125,136]. Ref. [125] reviewed 18 studies of the use of EMI for mapping soil moisture in which they reported that the correlation between ECa and soil moisture content ranged from 0.11 to 0.99. Whilst calibration and signal interpretation have been improved over recent years, EMI remains an indirect means of measuring soil moisture which varies greatly in time and space. Use of EMI techniques for routine measurement of soil moisture by farmers appears unlikely due to the time required for conducting surveys, the confounded nature of ECa response to soil properties, and the indirect relationship between ECa and soil moisture. 

### 4.3. Portable Optical Approaches (Vis-NIR, & NIR)

Optical approaches including vis-NIR, NIR and MIR have been successfully used to measure a wide range of soil properties including soil moisture [137,138,139,140]. vis-NIR (400–1000 nm) is generally regarded as the preferred approach for measuring soil properties including soil moisture due to its lower cost, greater portability and increased tolerance for sample preparation [141]. Within the vis-NIR range changes in spectral absorption result from thickening of water films on clay surfaces and capillaries. Several portable vis-NIR soil spectrometers have been commercialized, for measuring soil properties including soil moisture, including the NIRVascan ASP-NIR-350 M-Reflect (Allied Scientific Pro. QC, Canada) and NeoSpectra SWS62221 (Si-Ware Systems, Cairo, Egypt) which both require mobile phone connectivity for interpretation of spectral data.

Measurement of soil moisture using the NIR (800 nm–2500 nm) range is influenced by surface roughness, texture, soil pH, and clay content [142]. As such most recent NIR approaches compare readings from a select water sensitive band (1475 nm, 1979 nm, 1940 nm) to those of a water insensitive bands (1281 nm, 1314 nm, 1800 nm) in order to reduce the influence of other soil factors [142,143,144]. Furthermore, the depth of penetration of NIR is in the order of mm, such that samples can quickly change moisture content under ambient conditions. 

Soil moisture measurement by optical approaches (vis-NIR and NIR) for agricultural applications is limited by the need for large local calibration data sets and/or access to spectral libraries which is problematic in many agricultural regions due to poor data connectivity. Most optical soil sensors are also limited to surface applications, due to limited soil penetration. Whilst a number of mobile invasive optical sensors have been prototyped for mapping soil properties [145,146,147], these sensors suffer from issues with robustness, and as yet development has focused on nutrient rather than soil moisture measurement.

### 4.4. Microwaves and Ground Penetrating Radar (GPR)

Ground penetrating radar is a high-resolution, non-invasive technique routinely used for detecting buried infrastructure (pipes, wires, explosives) [25]. GPRs transmit high frequency microwaves in which the travel time between a radar transmitter and receiver is used to estimate the dielectric permittivity, $\in_{r}$ [3,12,15], according to,

(2) $$\in_{r}=\;{\left(\frac{c}{v}\right)}^{2}$$

where *c* is the speed of light in free space, and *v* is the velocity of the returning signal. GPR devices can be operated on the soil surface as traditional GPR surveys or as microwave or radar surveys from off-ground, or airborne platforms. On-ground GPR systems allow for deeper signals which are not prone to errors associated with surface soil conditions [3], whereas off-ground and airborne approaches are limited to shallower applications and are influenced by surface roughness and vegetation [148,149,150,151]. In the right conditions GPR can be a highly accurate means of ‘mapping’ soil moisture, for example [152] reported that the correlation between travel time and soil moisture ranged between 0.97 to 0.98. [153] report GPR average accuracy of ±1.5%, whilst [154] reported that estimation of soil moisture by GPR had an RMSE of 0.003 m^3^ m^−3^.

GPR is one of few, if not the only technique capable of accurately mapping spatial variations in soil moisture with both depth and distance. As such it would be expected that GPR would be widely adopted for use in agriculture. However, this review was unable to identify any commercial or non-research applications of GPR for mapping soil moisture in agriculture. Use of GPR for mapping soil moisture is slow, produces vast amounts of data, is prone to failure in saline and some clay soils, and requires a very high-end user knowledge to obtain good-quality data and valid interpretations [3,12,25,155]. Ref. [155] note that despite considerable advancement in GPR processing procedures, operation and interpretation of GPR data has not reached a level of maturity to be readily applied by nonexperts. 

### 4.5. Geographical Positioning Systems (GPS-IR, GNSS-IR)

Geographical positioning system interferometric reflectometry (GPS-IR or GNSS-IR) receivers use the difference between incoming and reflected L-band 1–2 GHz (15–30 cm wave length) microwaves to estimate the dielectric constant and thus soil moisture [156], in which increased soil moisture decreases the frequency and increases the noise and phase of the reflected signal [3,150]. 

The effective measurement depth of GPS-IR and GNSS-IR reflectometry is strongly influenced by wavelength, in which L band microwaves are only able to penetrate a few millimeters into moist soil, and up to around 7 cm in dry soil (usually reported as 5 cm), whilst the radius of the measured soil varies from about 50 m for an antenna installed at 1 m height, to 330 m for an antenna installed at 20 m height [157]. GPS-IR and GNSS approaches are reported to have RMSE values of around 0.035 cm^3^ cm^−3^ with measured samples [156,158], and correlations with commercially available soil moisture sensors of around 0.95 [158,159]. 

The primary benefit of GPS-IR and GNSS-IR approaches for use in agriculture lies in the fact that GPS signals are everywhere, and that measurement is possible with a commercially available handheld receiver with a modified antenna. Furthermore, GPS-IR and GNSS-IR receivers measure soil moisture over moderate to large distances (10 s–100 s of meters) depending on receiver height, which is very attractive for use in agriculture. Potential also exists to couple GPS-IR and GNSS-IR approaches with drones or UAV to conduct ‘on demand’ soil moisture surveys. GPS-IR and GNSS-IR approaches are subject to the same errors associated with other off-ground microwave-based approaches, namely; the shallow depth of measurement, soil roughness, satellite elevation angle, receiver height, leaf litter and vegetation biomass [150,151].

## 5. Discussion

With the exception of the neutron probe and cosmic ray sensors, use of soil moisture sensors for informing on farm decisions such as when and how much to irrigate, is greatly limited by the relatively small measured soil volume of most commercially available sensors (FDR, capacitance, gypsum block and granular matrix). Consider a farmer deciding when and how much to irrigate a 50-ha field based on the measurement of a sensor which senses as little as 10 cm^3^ soil, a measured to managed ratio of 1:10^10^. Use of existing soil moisture sensors requires a high degree of confidence that the sensor is correctly installed (i.e., no air gaps), that it is located in a representative soil type, that soil types are more or less uniform over an entire block, center pivot circle or management unit. One approach to overcome the measurement scale issue, is to use a large number of low-cost sensors. However, this is likely to require the cost of existing sensors to decrease by an order of magnitude or more, would greatly add to the navigational hazards for agricultural machinery, whilst the volume of data that would be created would pose considerable challenges for communication, storage and interpretation. Consequently, there is a considerable need to develop soil moisture sensors which can sense greater volumes of soil than existing sensors such that farmers can have more trust in sensor data. Ideally for agricultural applications, soil moisture sensors should measure soil moisture with elliptical sensing patterns at intermediate scales (i.e., 10 to 100 cm from the sensor), at discrete depth intervals (≤10 cm), over the whole root zone. They should be noninvasive or able to operate without close contact with the soil, use minimal power or require no external power, and operate equally in all soil types without soil specific calibration. No existing, new or emerging soil moisture sensor is able to meet all these requirements. For research purposes, soil moisture sensors need to be accurate, able to be used in a broad range of soil types including vertic and saline soils, have low to moderate power requirement for high frequency sampling and ideally have universal linear calibration. For invasive sensors, these requirements are largely met by existing 2nd generation TDR devices, and emerging advances in heat pulse and AHFO technology. However, for noninvasive research purposes, existing approaches are limited by issues with shallow penetration distance, moisture variable sensing depth, and cofounded soil factors such as salinity, texture, minerology, surface roughness.

Use of soil moisture sensors in agriculture is currently dominated by three technologies FDR, TDR and granular matrix matric potential sensors. Over the last decade relatively few improvements to these approaches or genuinely new means of measuring soil moisture have been commercialized. Notable exceptions include development of down hole TDR (Campbell Scientific), the reduced cost, size and complexity of TDR sensors (Acclima), development of TDR like approaches (Campbell Scientific, Delta T, ICT International, GroPoint) and cosmic ray sensors (Hydroinnova).

Review of the sensor and engineering literature demonstrates that a plethora of new, novel and emerging technologies for measuring soil moisture are under development. However, few have been commercialized or evaluated on farm. In order for emerging technologies to be adopted by farmers they need to overcome the many constraints experienced by existing sensor technology. Namely, the need for close soil–sensor contact, the small measured or sensed soil volume, need for multidepth measurements over the entire root zone, high sensor costs, power and communication requirements in remote locations, and hassle with installation and ability to move and relocate sensors.

Farmers require soil moisture sensors which read larger volumes of soil than is currently available with existing techniques in order to have to make data-based decisions on irrigation management. Currently, the only sensors able to measure soil moisture at intermediate scales (10–100 cm from the sensor) is the neutron probe which has gone out of favor due to regulatory issues. Emerging approaches with the capacity to measure soil moisture at intermediate or larger scales include AHFO and paired transceiver approaches. Development of paired transceiver approaches is currently limited by inadequate theoretical understanding of seismic, acoustic and radio wave propagation through variably saturated soil and the cofounded relationship between signal velocity and attenuation with a range of soil properties other than moisture.

Multidepth probe configurations are popular amongst farmers as multi-depth probes can be used to estimate irrigation requirement, irrigation timing and the depth to which roots are extracting soil moisture. Multi-depth or down hole soil moisture sensors are dominated by FDR technology, in which downhole TDR has only recently been commercialized. Options exist to reconfigure single depth invasive sensors into multi depth probes. Specifically, the simplified manufacture of heat pulse sensors combined with use of lowcost on-board controllers should enable development of competitively priced, multi-depth heat pulse probes which in theory should be more accurate, and less prone to errors associated with salinity, temperature and poor soil–sensor contact than current FDR multi-depth probes. Reconfiguration of AHFO, heat pulse, paired transceiver and possibly invasive open-ended microwave approaches into multi-depth probes also appears achievable.

Opportunities for further development of mobile on-ground or off-ground proximal soil moisture sensors which are able to determine soil moisture at multiple depths seems limited, with few if any emerging new approaches. Emerging noninvasive approaches such as GNSS-IR, and most other microwave-based approaches are greatly limited by the shallow penetration depth of L-band microwaves. Furthermore, adoption of existing approaches such as EMI and GPR is unlikely, even with advances in data interpretation due to the time and effort required to conduct surveys, expertise required and cofounded nature of the data.

The cost of sensors and data communication remains a considerable impediment to the adoption of soil moisture monitoring in agriculture. Significant reductions in the cost of FDR and TDR sensors has been achieved in recent years, whilst improvements in manufacturing also promise to reduce the cost of heat pulse technology in the near future. Currently the only truly low-cost approach is RFID in which sensors could be as little as < USD 1. However, RFID is limited by its shallow depth of penetration and need for readers to be within proximity of the sensor. Summary of sensor types, limitations and research priorities are presented in Table 1. 

## 6. Conclusions

Farmers have never had the technology that they want to be using for measuring soil moisture. Instead they have made use of the technology which has been made available to them, namely TDR, FDR and granular matrix sensors. Review of the literature reveals a plethora of improved, novel and new approaches for measuring soil moisture, in which agriculture is almost always identified as a potential end user of the technology. However, few studies demonstrate understanding of how emerging sensor technology may overcome constraints associated with the use of existing soil moisture sensors or acknowledge in what soil or agricultural systems emerging technology is suited or limited.

Future development of soil moisture sensors for use by farmers in agriculture would greatly benefit from greater cooperation between sensor engineers, soil scientist, and agriculturalists in order to develop, new, useful, usable soil moisture sensors that overcome the constraints to the use of existing soil moisture sensors. New sensor technologies need to pay greater attention to overcoming logistical constraints imposed by agriculture including frequent tillage, operation in remote locations and limited technical skills of users, as well as the need to increase the volume of sensed soil without losing specific depth information. In addition, new approaches need to be developed for use in stony, vertic and saline soils. This remains a considerable challenge, in which no single, new, novel or emerging technology is a clear winner.

## Figures and Tables

**Table 1:** Summary of selected existing, novel and new types of soil moisture sensing technologies.

	Accuracy & reliability	Installation	Measurement scale	Development stage	Suitable soil / Agriculture	Cost	Key Limitations	Key Advantages	Research Needs	Reference
Cosmic Ray	High	NI	Very large	Commercialised	All	High	Variable measurement area and depth	Large measurement scale	Calibration algorithms	[1]
Downhole TDR	High	D	Small	Commercialised	Non stony, non highly vertic soils	Moderate	Requires access hole	Larger measured volume and less affected by soil contact than FDR	Evaluation of usability	[36,38]
Low cost FDR	Poor, variable	I	Very small	Prototype to Commercialised	Non stony, non vertic, non saline soils. Shallow rooted crops	Low	Soil-sensor contact, salinity, temperature	Low cost, mass production	Evaluation of performance	[41,42,49]
RFID	Moderate (unknown)	NI, OG	Surface only, small area	Prototype / Conceptual	Most soils (unknown) Nurseries, glasshouse, very shallow rooted crops	Very low	Shallow depth, requires active reader	Very low cost	Identify suitable applications, simplify readings	[50,52,53,160]
GPS-IR & GNSS-IR	Moderate, (unknown)	NI, OG, M	Surface only, large area	Early prototype	Most (unknown). Shallow rooted crops	Low	Shallow depth of measurement, soil roughness	Available everywhere, intermediate scale, can be stationary or mobilised	Signal analysis	[150,156,158,160]
GPR	Moderate to high	NI, M, OG	Medium & depth-wise	Advanced	Most soils except saline and some clays	Moderate - high	Expertise required for analysis	Mobile, extend to several metres depth	Algorithms for improved estimation of soil moisture	[153,154,161]
Paired radio / acoustic / seismic waves	Unknown, (soil specific)	I, D	Unknown, medium	Early Prototype / Conceptual	Unknown, less successful in saline, compacted soils	Low - moderate	Unknown effect of soil properties on signal attenuation	Medium scale of measurement Completely buried	Improved theoretical understanding of wave propagation in soil, multi-wave analysis of soil properties and soil moisture.	[61,70,76,162]
Seismoelectric	Unknown	NI	Medium - large	Early	Unknown	Unknown	Limited understanding of streaming current behavior in soils	Ability to simultaneously measure, porosity, hydraulic conductivity and moisture content in 2D sections	Downscaling, theoretical understanding, application, evaluation in agricultural soils	[77,79,82,84,85]
EMI	Variable	NI, M OG	Medium	Commercialised	Most non saline, non ferric soils	Moderate	Bulked signal, need for local calibration	Mobile, affordable, moderate operation and data analysis skills	Machine learning based analysis	[119,125,126]
Nir VIS, NIR, MIR	High	OG	surface	Commercialised	All	High	Shallow depth of penetration. Sample preparation	Quick, relatively straight forward, non invasive	Robustness or below ground applications	[142,143,147]
Heat Pulse	High	I	Small	Commercialised	Most, preferably non stony and non vertic	Moderate	Power usage, costly electronics	More accurate and larger measurement area than FDR. Not influenced by salinity	Lower production cost	[89,92]
Thermo-Optical Fiber DTS	High	I, D	1–5 cm × 1000 m	Prototype	Non vertic soils, drip irrigation, perennial tree crops	Unknown	Fragility of the optic fiber, requires good soil contact	Distributed approach with mm accuracy positioning	Sensor robustness, evaluation in agricultural soils	[96,106]
HCT	High	I	1–5 cm	Prototype	Non vertic, and non saline	Unknown	Complicated de-airing	Measurement range 0 to −1500 kPa	Simplified deairing and filling apparatus, new design concepts.	[22,23,147]
Hydrogels	Unknown	I, D	1–5 cm	Prototype / conceptual	Non vertic and potentially non saline soils	Unknown	Soil – sensor contact, effects of pH, and gel lifespan	Potentially low cost, larger measurement range than tensiometers	Field evaluation, new compounds, application design	[110,111,112]

NI—noninvasive, D down access hole or tube, I invasive, M mobile, OG off ground.
